# Inferior Mesenteric Artery Injury in Post-lumbar Microdiscectomy: A Case Report

**DOI:** 10.7759/cureus.42998

**Published:** 2023-08-05

**Authors:** Abdullah K Aljohani, Mohammed Khalid Bin Yunus, Albaraa A Fallatah, Omar M Kheder, Kinan S Almolki, Hani Alawad, Rayan Talal Halawani, Abdelsalam A Abdelaziz, Ahmed Sherif

**Affiliations:** 1 Medicine and Surgery, Taibah University, Medina, SAU; 2 Medicine, Taibah University, Medina, SAU; 3 Neurosurgery, King Fahad Hospital, Medina, SAU; 4 Interventional Radiology, King Fahad Hospital, Medina, SAU

**Keywords:** lumbar microdiscectomy, vascular injury, lumbar disc herniation, complication, inferior mesenteric artery

## Abstract

Iatrogenic vascular injury during lumbar microdiscectomy is a rather rare complication, but it can have fatal consequences. Here, we report a patient who underwent an L5-S1 microdiscectomy, which was complicated by inferior mesenteric artery injury. The patient presented in the recovery room with symptoms of hypotension and tachycardia after the operation which was successfully managed by endovascular embolization. The patient was positioned in a prone position, which may have contributed to the development of vascular injury. To prevent potential complications, we advised using the Jackson table rather than a standard surgical table and thoroughly inspecting the abdomen and pelvis prior to the operation.

## Introduction

Lumbar microdiscectomy, among different surgical approaches, is considered the gold standard for treating lumbar disc herniation so far, and it is known as a low-risk procedure. However, complications can occur, such as nerve root injury, wound complications, and recurrent disc prolapse [1,2]. Iatrogenic vascular injury is rare, with an estimated incidence rate of less than 0.05% [3-5]. However, it can lead to fatal outcomes if the diagnosis and management are started late [6]. The clinical presentation depends on the type of injury, symptoms, and signs that occurred during or shortly after the operation, such as hypotension (77%), tachycardia, wide pulse pressure (53%), and abdominal distention (20%), usually due to vascular laceration, which is the first significant indicator of iatrogenic injury and can give rise to a 10% mortality rate, further increasing to 38% if the injured artery is the aorta [7]. Conversely, delayed onset symptoms are related to the formation of an arteriovenous fistula (AVF) and pseudoaneurysm; however, sometimes the presentation of AVF can emerge acutely [7,8]. The site of vascular injury is mainly associated with the level of the disc, so approximately half of the vascular injury events during lumbar discectomy occurred at the level of L4/L5, mainly associated with iliac vessel injury [7]. Cases of transected inferior mesenteric artery are considered unique due to their rarity [9].

In our study, we reviewed a case of vascular injury of the inferior mesenteric artery during lumbar microdiscectomy at the level of L5-S1 with an early post-operative presentation.

## Case presentation

Our patient, a 45-year-old obese woman with a medical history of hypothyroidism, presented to the outpatient department of neurosurgery at King Fahad Hospital, Medina, in March 2021 after three months of falling and complaining of chronic back pain and right sciatica.

**Figure 1:**
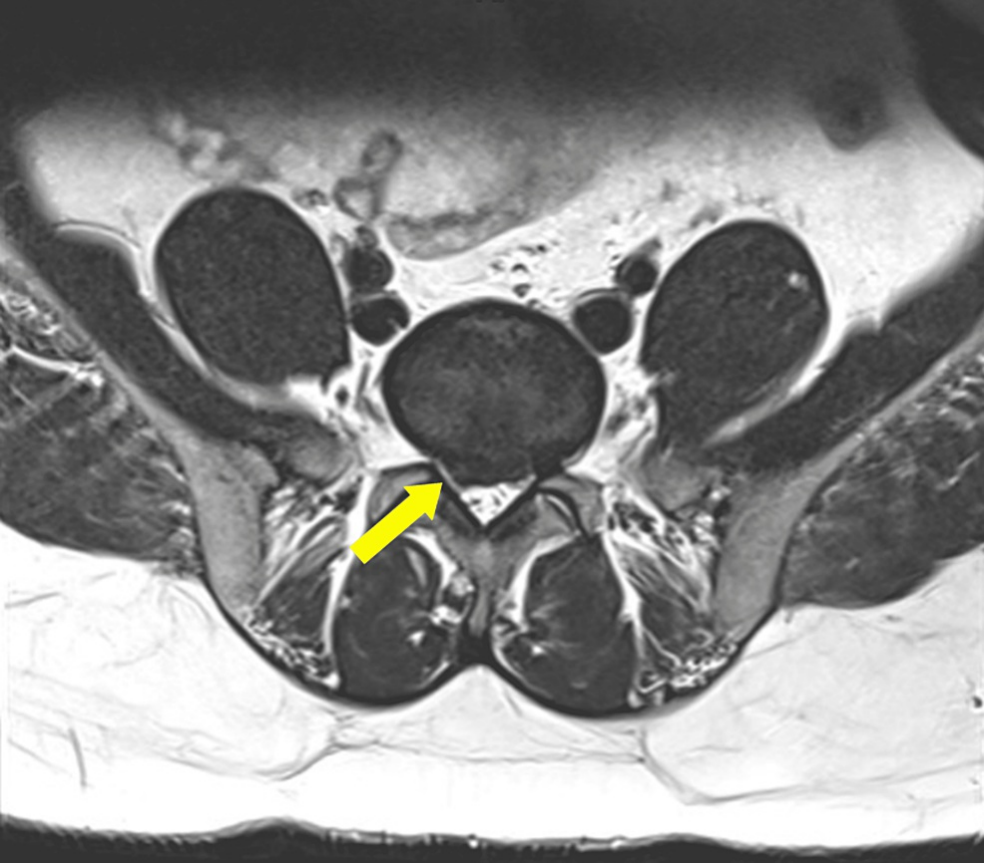
L5/S1 disc herniation right posterolateral compressing opposing thecal aspect and neural recess and impinging both nerve roots, especially on the right.

Examinations revealed no focal motor impairment, normal muscle power (5/5 on the scale), and normal joints in the upper and lower limbs. The straight leg raise test was positive at 65 degrees, indicating right sciatica. She had no sores or edema and was able to walk normally. However, she experienced hypoesthesia in her right L5/S1 dermatomes and hyporeflexia in her ankle reflex. On investigations, the MRI showed L5/S1 disc herniation right posterolaterally compressing the thecal aspect and neural recess and impinging on both nerve roots, especially on the right (Figure 1).

She had been given analgesics and was given a referral to a physiotherapy and rehabilitation center. However, after the follow-up, her pain persisted. As there had been no change in her condition with conservative treatment, surgery was advised, and the patient agreed and was prepared for the surgical procedure (microdiscectomy). 

She was placed on the surgical table (Trumpf TruSystem 7000 Mobile Surgical Table, Motorized Base {MB}; Charleston, SC: Trumpf Medical Systems, Inc.) in a prone position in the operating room while under general anesthesia (GA). A C-arm x-ray confirmed the level. The right paraspinal muscle was dissected after a 4 cm skin incision with satisfactory hemostasis. The L5/S1 disc space is visible, followed by the lower lamina 5 fenestration with the upper S1. When the dura was dissected, the flavum ligament was gently removed. Then, the nerve root was observed and foraminotomy was performed. After that, the disc bulge was exposed, and the nerve root was gently retracted using a nerve root retractor. Furthermore, the disc was removed using a pituitary disc rongeur. Following the discectomy, 80 mg of methylprednisolone was administered locally before closing the muscle fascia and applying a sterile dressing. The patient was then extubated and shifted to the recovery area.

**Figure 2:**
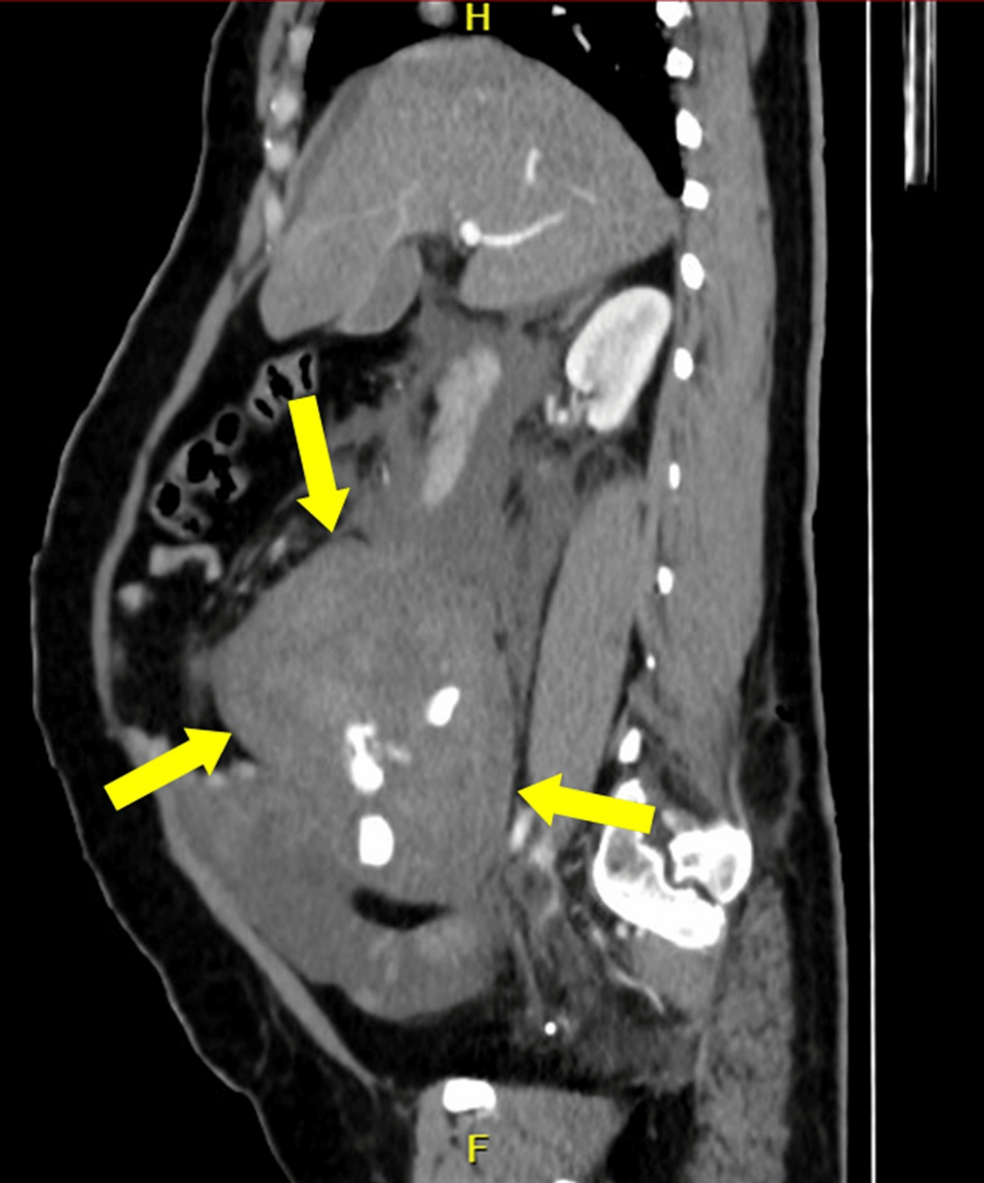
Vascular extravasation and large retroperitoneal hematoma on CTA (sagittal view). CTA: computed tomography angiography

**Figure 3:**
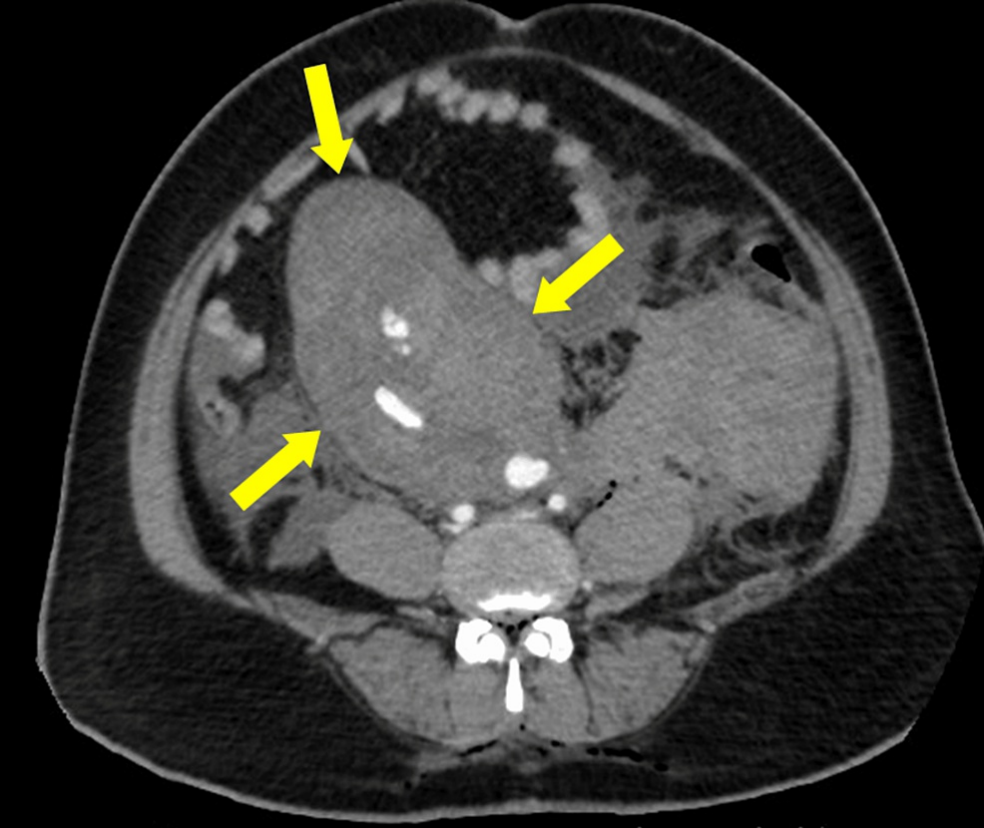
Vascular extravasation and large retroperitoneal hematoma on CTA (axial view). CTA: computed tomography angiography

Unfortunately, the patient became hypotensive and tachycardic in the recovery room and subsequently went into shock. Her abdomen was distended, and investigations found that her hemoglobin was 7.1 g/dL and hematocrit was 32%. Urgent abdominal CT and CT angiography revealed vascular extravasation and a large retroperitoneal hematoma that accumulated within about 5 hours after surgery (Figures 2, 3).

**Figure 4:**
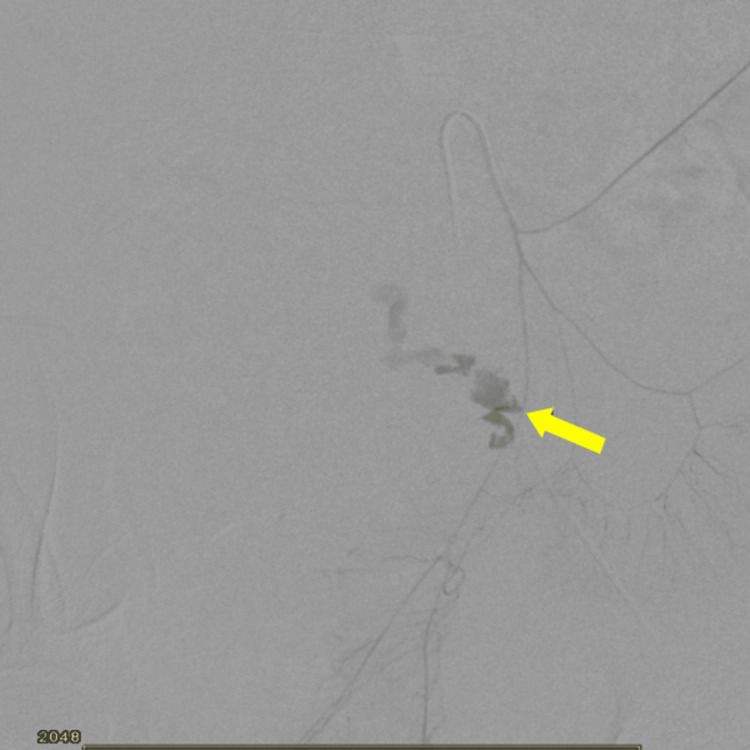
Inferior mesenteric artery area extravasation on DSA. DSA: digital subtraction angiography

**Figure 5:**
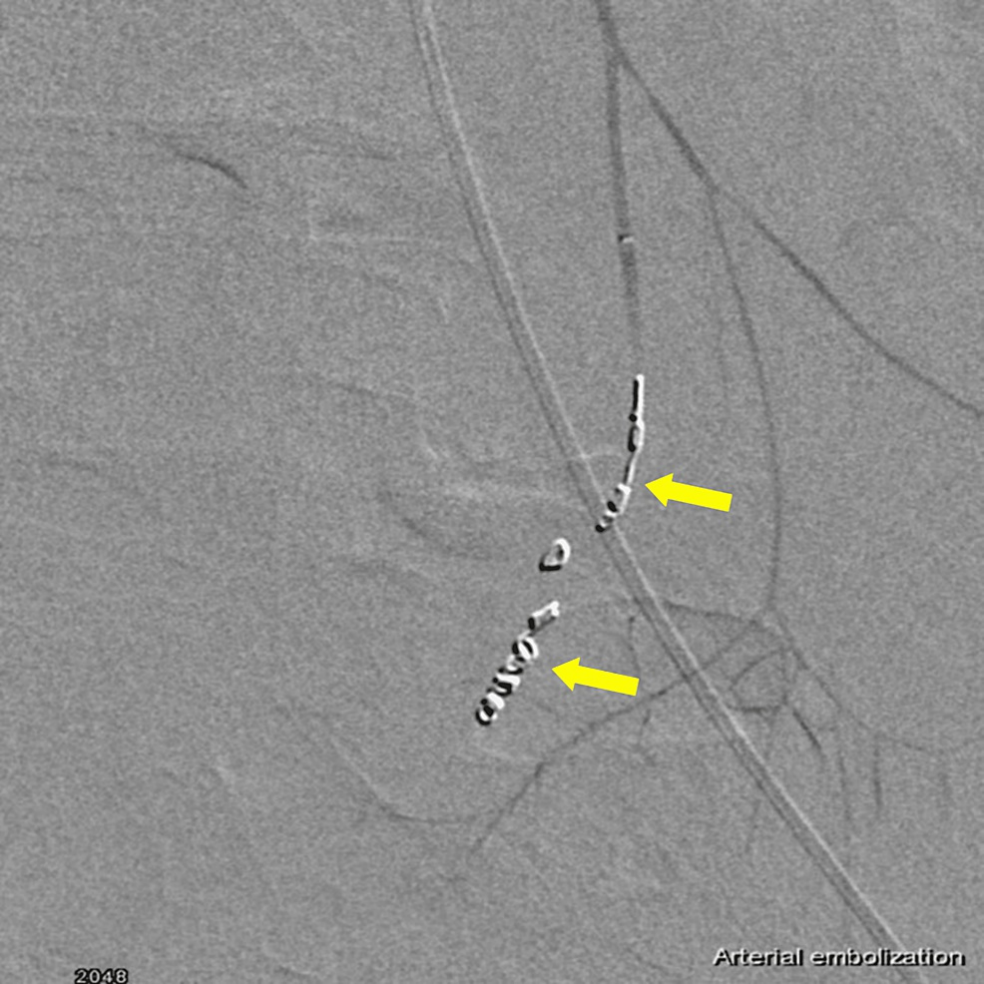
Inferior mesenteric artery area embolization.

After that, vascular surgery and interventional radiologists were urgently consulted, and digital subtraction angiography (DSA) was performed, which showed inferior mesenteric artery extravasation (Figure 4). Endovascular embolization was successfully performed in the same sitting using multiple 2, 3, and 4-mm pushable 0.018-inch coils (Bloomington, IN: Cook Medical LLC) (Figure 5).

She received a total of seven units of packed red blood cells (PRBC), six units of fresh frozen plasma (FFP), and six units of platelets. An endotracheal tube (ETT) size 7 was connected to a mechanical ventilator, air conditioning and mechanical ventilation (ACMV) system, with a central line connected to ongoing Levophed at 6 μg/h. Then, she was shifted to the surgical intensive care unit (SICU). After stabilization, the patient was shifted to the ICU. Two weeks later, she was transferred to a regular ward and discharged from the hospital with a healthy discectomy wound and neurologically intact.

## Discussion

Iatrogenic vascular injuries during spine surgery are rare but life-threatening, with a total fatality rate ranging from 15-65%. These injuries may not always be evident in the clinical setting and depend on several variables, including the surgical method, the mechanism of the injury, and the vessel's caliper. They could occur during, or following surgery [10].

In the prone position during lumbar spine surgery, significant clinical bleeding can occur, involving both arterial and venous blood vessels. The importance of avoiding abdominal compression to reduce the risk of bleeding has long been recognized, leading to the utilization of various methods to support patients in the prone position during surgery. The surgical approach and the number of spinal levels involved can also affect the amount of blood lost [11].

Our patient, a 45-year-old obese woman operated for right posterolateral L5/S1 disc herniation. The patient was subjected to surgery and developed hypotension and tachycardia post-surgery. The screening revealed intra-abdominal hematoma secondary to vascular injury, and early embolization was done for inferior mesenteric artery injury. It is proposed that the patient's position in the prone position and obesity may have contributed to the significant vascular injury during microdiscectomy [12].

The "tuck" position, described by Wayne in 1967, showed the lowest vena caval pressure of any position studied. This posture was particularly beneficial for obese participants as it significantly reduced caval pressure. Direct pressure on the major arteries in the abdomen can make them more susceptible to injury, pushing them against the vertebral column [13]. Hence, vascular injury could have occurred during the removal of disc materials using disc forceps (rongeur), as the inferior mesenteric artery injury could be fixed over the anterior wall of the disc level.

The Jackson table was found to be efficient in supporting patients of various body sizes and shapes, reducing elevated intra-abdominal pressure as a significant factor [11]. Another method, putting an overweight patient in the lateral position, is less familiar to most surgeons.

In 1945, Robert and Paul reported the first case of vascular injury after lumbar discectomy, resulting in arteriovenous fistula (AVF) formation [14]. Since then, a significant number of additional cases of vascular injury during lumbar spinal surgery have been recorded. Vascular tears are the most frequent type of injury, and they can manifest in a variety of ways, from profuse bleeding to early post-operative shock symptoms, such as tachycardia and hypotension [15].

Birkeland and Taylor reported a unique case in which the inferior mesenteric artery had been transected at its junction with the superior rectal artery [9]. Kim et al. reported a case of lumbar microdiscectomy related to iatrogenic vascular damage and noted that the left common iliac arteries are the most often affected vessels, as they are situated directly anterior to the disc area [16]. Keskin et al. also reported three cases of iatrogenic vascular injury, all of which had left common iliac artery injuries, and two of them had hypotension and tachycardia [17].

This study emphasizes the importance of inspecting for any further bleeding after a microdiscectomy, the use of the Jackson table to assist patients of varied body sizes and shapes, the elimination of raised intra-abdominal pressure, and the importance of close observation during recovery and post-operation periods. Timely management is vital in the proper management of this rare and fatal complication.

## Conclusions

Iatrogenic vascular injury during lumbar discectomy is a rare but potentially fatal consequence. When a patient experiences hypotension, tachycardia, and abdominal distention following a lumbar discectomy, early CTA is recommended to detect the source of bleeding, and early intervention is important to control the hemorrhage. We also recommend properly checking the abdomen and pelvis before the operation and using the Jackson table instead of an ordinary surgical table to avoid possible complications.

## References

[1] Ruan W, Feng F, Liu Z, Xie J, Cai L, Ping A (2016). Comparison of percutaneous endoscopic lumbar discectomy versus open lumbar microdiscectomy for lumbar disc herniation: a meta-analysis. Int J Surg.

[2] Shriver MF, Xie JJ, Tye EY, Rosenbaum BP, Kshettry VR, Benzel EC, Mroz TE (2015). Lumbar microdiscectomy complication rates: a systematic review and meta-analysis. Neurosurg Focus.

[3] Szolar DH, Preidler KW, Steiner H (1996). Vascular complications in lumbar disk surgery: report of four cases. Neuroradiology.

[4] Bingol H, Cingoz F, Yilmaz AT, Yasar M, Tatar H (2004). Vascular complications related to lumbar disc surgery. J Neurosurg.

[5] Denli Yalvac ES, Balak N (2020). The probability of iatrogenic major vascular injury in lumbar discectomy. Br J Neurosurg.

[6] van Zitteren M, Fan B, Lohle PN, de Nie JC, de Waal Malefijt J, Vriens PW, Heyligers JM (2013). A shift toward endovascular repair for vascular complications in lumbar disc surgery during the last decade. Ann Vasc Surg.

[7] Papadoulas S, Konstantinou D, Kourea HP, Kritikos N, Haftouras N, Tsolakis JA (2002). Vascular injury complicating lumbar disc surgery. A systematic review. Eur J Vasc Endovasc Surg.

[8] Erkut B, Unlü Y, Kaygin MA, Colak A, Erdem AF (2007). Iatrogenic vascular injury during to lumbar disc surgery. Acta Neurochir (Wien).

[9] Birkeland IW, Taylor TK (1969). Major vascular injuries in lumbar disc surgery. J Bone Joint Surg Br.

[10] Giotta Lucifero A, Gragnaniello C, Baldoncini M (2021). Rating the incidence of iatrogenic vascular injuries in thoracic and lumbar spine surgery as regards the approach: a PRISMA-based literature review. Eur Spine J.

[11] Mathai KM, Kang JD, Donaldson WF, Lee JY, Buffington CW (2012). Prediction of blood loss during surgery on the lumbar spine with the patient supported prone on the Jackson table. Spine J.

[12] Kwee MM, Ho YH, Rozen WM (2015). The prone position during surgery and its complications: a systematic review and evidence-based guidelines. Int Surg.

[13] Wayne SJ (1967). The tuck position for lumbar-disc surgery. J Bone Joint Surg Am.

[14] Robert RL, Paul DW (1945). Arteriovenous fistula between the right common iliac artery and the inferior vena cava. JAMA Surg.

[15] Huttman D, Cyriac M, Yu W, O'Brien JR (2016). The unusual presentation of a vascular injury after lumbar microdiscectomy: case report. J Neurosurg Spine.

[16] Kim DH, Kim TW, Kim MK, Park KH (2016). Iatrogenic vascular injury occurring during discectomy in a spondylodiscitis patient. Korean J Neurotrauma.

[17] Keskin M, Serin KR, Genc FA, Aksoy M, Yanar F, Kurtoglu M (2013). Iatrogenic major vascular injury during lumbar discectomy: report of three cases. Turk Neurosurg.

